# Prevalence of Blood-Borne Viral Infections among Blood Donors of Tripura

**DOI:** 10.5005/jp-journals-10018-1106

**Published:** 2014-07-28

**Authors:** Pradip Bhaumik, Kalyan Debnath

**Affiliations:** 1 Department of Medicine, Agartala Government Medical College, Agartala, Tripura, India; 2 Indira Gandhi Memorial Hospital, Agartala, Tripura, India

**Keywords:** Blood donors, Hepatitis B virus, Hepatitis C virus, Human immunodeficiency virus.

## Abstract

**Background:**

Blood-borne viral infections, like hepatitis B virus (HBV), hepatitis C virus (HCV) and human immunodeficiency virus (HIV), are most common during blood transfusion. Morbidity and mortality resulting from the transfusion of infected blood have far reaching consequences not only for the recipients themselves but also for their families, communities and the wider society.

**Aims:**

The study was designed to determine the prevalence of HBV, HCV and HIV among voluntary and replacement blood donors of Tripura, India, and to study the trends of HBV, HCV and HIV infections in the population.

**Materials and methods:**

This study is a retrospective cross-sectional study. The data was collected for consecutive 8 years from 2005 to 2013. Analyses were done in respect of total blood collection and HBV, HCV and HIV infections among the donors.

**Results:**

Among all donors, 91.8% was voluntary donors and 8.2% was replacement donors. The average HBV, HCV and HIV positivity was 1.2% (95% CI: 1.155-1.255), 0.109% (95% CI: 0.0950.125) and 0.093% (95% CI: 0.080-0.108) respectively. Among these, HBV seropositivity was 1.19% among voluntary donors and 1.33% among replacement donors and, in case of HCV and HIV, the seropositivity among voluntary and replacement donors were 0.109%, 0.11% and 0.089%, 0.145% respectively. HBV positivity was reduced in 8 years, whereas those of HCV and HIV remain unchanged.

**Conclusion:**

The most important observation of this study is gradual decrease in prevalence of HBV (p = 0.0018), whereas change in prevalence of HCV and HIV was not statistically significant. This might be due to mass hepatitis B vaccination program in Tripura.

**How to cite this article:** Bhaumik P, Debnath K. Prevalence of Blood-Borne Viral Infections among Blood Donors of Tripura. Euroasian J Hepato-Gastroenterol 2014;4(2):79-82.

## INTRODUCTION

Blood transfusion has been an integral and life-saving procedure of modern medical science, since the discovery of human whole blood transfusion in 1818 by Dr James Bundell. But, unsafe transfusion practices put millions of people at risk of transfusion transmissible infections (TTIs). Despite the availability of improved donor screening technologies and viral inactivation procedures, the risk of TTIs still remains a major concern. Factors, such as blood donation during window period, emergence of newer transmissible pathogens and prevalence of asymptomatic carriers pose a serious challenge to blood safety.^1^ Blood-borne viral infections like hepatitis B virus (HBV), hepatitis C virus (HCV) and human immunodeficiency virus (HIV) are the most common during blood transfusion. Globally, there are approximately 400 million HBV carriers in the world and India is in the intermediate zone of prevalence (2-7%)^2^ and burden of HCV is around 170 million.

The use of unscreened HBV-infected blood and blood products will result in the transmission of HBV in the vast majority of cases. The distinction between acute and chronic infection is not relevant to blood screening; all HBsAg positive donations should be considered to be at high risk of transmitting HBV and should not be released for transfusion. Additionally, some studies indicate that even when HBsAg is negative, some individuals may have low levels of detectable viral deoxyribonucleic acid (DNA), which will be transmitted by blood and may cause infection in the recipients.^3,4^

There are 2.39 million people living with HIV or acquired immunodeficiency syndrome (AIDS) in India with an estimated adult prevalence of 0.31%^5^ and transmission due to blood and blood products is around 1%. HIV can be present in the bloodstream in high concentrations and it is stable at the temperatures at which blood and individual blood components are stored. Infectivity estimates for the transfusion of infected blood products are much higher (around 95%) than for other modes of HIV transmission owing to the much larger viral dose per exposure than for other routes.^6^ The prevalence of TTIs in voluntary non-remunerated blood donors is generally much lower than among family, replacement^7-9^ and paid donors.^10-12^

Morbidity and mortality resulting from the transfusion of infected blood have far reaching consequences not only for the recipients themselves but also for their families, communities and the wider society. Hence, the current study is to estimate the prevalence of blood-borne viral infections among voluntary and replacement donors of Tripura. We also evaluated the trend of these infections over a period of 8 years.

## MATERIALS AND METHODS

This study is a retrospective cross-sectional study. Tripura is a state in India, which is famous for its voluntary blood donation system. For last few years, almost 95% of the blood collected in the various blood banks of Tripura is mainly from voluntary blood donation. At present, Tripura has got six blood banks and all are under direct administrative control of Tripura State Blood Transfusion Council. All the blood banks screen every unit of collected blood for three blood-borne viruses, i.e. HBV, HCV and HIV along with malaria and venereal disease research laboratory (VDRL). The central records are collaterally maintained by Tripura State Blood Transfusion Council and Tripura State AIDS Control Society. The study was planned to collect blood transfusion data and records of blood-borne virus infection among blood donors.

The blood donors are of two types: voluntary donors who donate blood as a part of social responsibility and replacement donors donate blood on exchange system for their nearest and dearest ones. The data was collected for consecutive 8 years from 2005 to 2013. The analysis was done in respect of total blood collection and HBV, HCV and HIV infections among the donors. The data could not be accumulated on the basis of age, sex and repeat donations as this is a retrospective study. The data were analyzed for:

- Percentage of voluntary donation in comparison to replacement donation.
- Percentage of HBV, HCV and HIV among blood donors.
- The significance of trend of infection was analyzed by linear regression, and the values were correlated with the population of Tripura.

## RESULTS

The study conducted for 8 years period from 2005 to 2013. Among the donors, 91.8% was voluntary donors and 8.2% was replacement donors. The average of HBV, HCV and HIV positivity in 8 years period was 1.2% (95% CI: 1.155-1.255), 0.109% (95% CI: 0.095-0.125) and 0.093% (95% CI: 0.0800.108) respectively. Among these, HBV seropositivity was 1.19% among voluntary donors and 1.33% among replacement donors and in case of HCV and HIV the seropositivity among voluntary and replacement donors were 0.109%, 0.11°%, 0.089%, 0.145% respectively.

The year-wise distribution of HBV, HCV and HIV positivity in the study group has been given in Table 1.

**Table 1:** **Table 1:** Serial observation of blood-borne viruses among blood donors

*Year*		*Total donor*		*Total HBV positive*		*HBV* *positive* *(%)*		*95% CI*		*Total* *HCV* *positive*		*HCV* *positive* *(%)*		*95% CI*		*Total* *HIV* *positive*		*HIV* *positive* *(%)*		*95% CI*	
2005-06		17,382		276		1.59		1.404-1.776		25		0.14		0.084-0.196		17		0.09		0.051-0.144	
2006-07		19,266		244		1.27		1.112-1.428		19		0.09		0.048-0.132		36		0.19		0.129-0.251	
2007-08		21,644		305		1.41		1.253-1.567		7		0.03		0.007-0.053		17		0.08		0.050-0.130	
2008-09		24,195		317		1.31		1.167-1.453		27		0.11		0.068-0.152		19		0.08		0.044-0.116	
2009-10		22,736		280		1.23		1.087-1.373		54		0.24		0.177-0.303		18		0.08		0.043-0.115	
2010-11		23,867		274		1.15		1.015-1.285		29		0.12		0.076-0.164		27		0.11		0.070-0.156	
2011-12		22,744		246		1.08		0.946-1.214		20		0.09		0.049-0.125		13		0.06		0.026-0.088	
2012-13		25,468		194		0.76		0.654-0.866		14		0.05		0.026-0.084		19		0.07		0.041-0.109	

HBV: Hepatitis B virus; HCV: Hepatitis C virus; HIV: Human immunodeficiency virus

**Graph 1:**
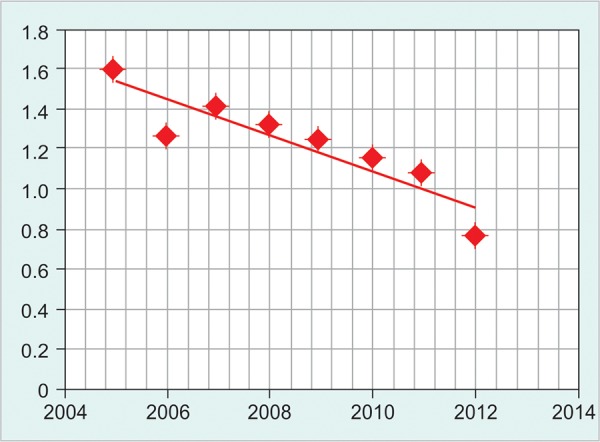
Kinetics of hepatitis B virus

**Graph 2:**
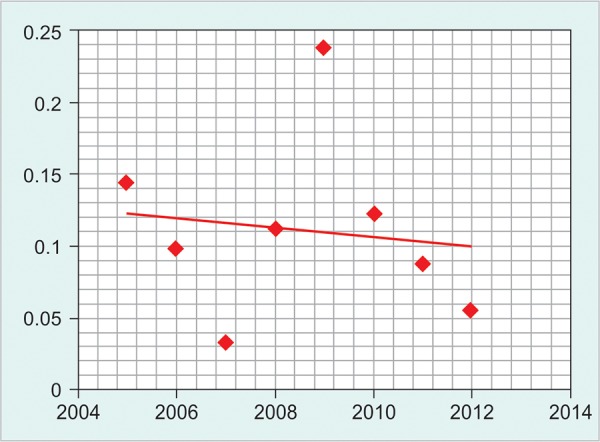
Kinetics of hepatitis C virus

**Graph 3:**
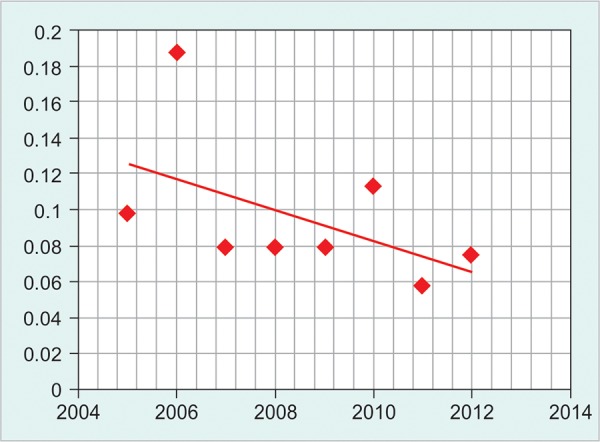
Kinetics of human immunodeficiency virus

There is a gradual fall of HBV prevalence among blood donors as shown in Graph 1. However, the pattern of HCV and HIV prevalence did not show a homogenous pattern (Graphs 2 and 3).

## DISCUSSION

The study reflects total samples screened during 8 years, although possibility of repeat donation could not completely excluded in this study. Average yearly donation was 22,163 and, out of this, 91.8% was from voluntary donation and 8.2% was from replacement donation. The percentage of voluntary blood donation was much higher in comparison to other states of India. The percentage of voluntary and replacement donation in Andhra Pradesh is 41.64% and 68.36%^13^, in Mangalore, it is 61.2% and 38.8%^14^ and, in Chandigarh, it is 45% and 55%^15^ respectively.

The fall in prevalence of HBV among blood donors was statistically significant (p = 0.0018). The overall HBV prevalence at Tripura was 1.2% during 8 years study, which is relatively lower than other parts of India; 1.66% in West Bengal,^16^ 1.7% in Haryana,^17^ 3.44% in Western India and^18^ 1.96% in Lucknow.^19^ Whereas in California, USA, the prevalence of HBV among first time donors was 0.28% and in USA r isks of HBV transmission among blood donors is extremely low 1 in 63,000.^20-22^ replacement donors have got higher prevalence in comparison to voluntary donors like other parts of the country, such as in Karnataka, HBV seropositivity is 0.65% in replacement donors and 0.42% in voluntary donors and, in Lucknow, it is 1.67% and 0.24% respectively.^23,24^

The HCV prevalence was neither different over the years, nor was it significantly different among voluntary donors and replacement donors.

The average prevalence of HIV among blood donors was 0.093% (95% CI: 0.080-0.108) for voluntary donors it was 0.089% and whereas it was 0.145% among replacement donors. Pallavi et al reported HCV and HIV prevalence of 0.23% and 0.44% among blood donors from Mysore, India,^25^ and Pathak et al reported 0.7% and 0.25% of HCV and HIV seroprevalence among blood donors in a tertiary care hospital in Delhi.^26^

On evaluating the cause of decrease in prevalence of HBV in Tripura, it is observed that, since 2003, Hepatitis Foundation of Tripura, a social organization, is campaigning and organizing mass Hepatitis B Vaccination Program in Tripura. They have achieved vaccination of about 30% of population. This might have got an impact on decrease in HBV prevalence in Tripura.

Prevalence of blood borne virus among blood donors is a reflection of disease in a community. HBV prevalence is in gradual regression in Tripura, which can be correlated with the mass vaccination program in the state organized by a social organization Hepatitis Foundation of Tripura in association with State Government of Tripura. As a positive response has been found about HBV prevalence by integrated efforts of health policy implementation, more works need to be accomplished to contain HCV and HIV in Tripura.
